# Fabrication of FeAl Intermetallic Foams by Tartaric Acid-Assisted Self-Propagating High-Temperature Synthesis

**DOI:** 10.3390/ma11040621

**Published:** 2018-04-18

**Authors:** Krzysztof Karczewski, Wojciech J. Stępniowski, Marco Salerno

**Affiliations:** 1 Department of Advanced Materials and Technologies, Faculty of Advanced Technology and Chemistry, Military University of Technology, Urbanowicza 2 Str., 00-908 Warszawa, Poland; krzysztof.karczewski@wat.edu.pl (K.K.); wos218@lehigh.edu (W.J.S.); 2 Department of Materials Science & Engineering, Loewy Institute, Lehigh University, 5 East Packer Avenue, Bethlehem, PA 18015, USA; 3 Materials Characterization Facility, Istituto Italiano di Tecnologia, via Morego 30, 16163 Genova, Italy

**Keywords:** porous intermetallics, sintering, chemical-compound-assisted sintering, tartaric acid, Fe-Al binary diagram

## Abstract

Iron aluminides are intermetallics with interesting applications in porous form thanks to their mechanical and corrosion resistance properties. However, making porous forms of these materials is not easy due to their high melting points. We formed FeAl foams by elemental iron and aluminum powders sintering with tartaric acid additive. Tartaric acid worked as an in situ gas-releasing agent during the self-propagating high-temperature synthesis of FeAl intermetallic alloy, which was confirmed by X-ray diffraction measurements. The porosity of the formed foams was up to 36 ± 4%. In the core of the sample, the average equivalent circle diameter was found to be 47 ± 20 µm, while on the surface, it was 35 ± 16 µm; thus, the spread of the pore size was smaller than reported previously. To investigate functional applications of the formed FeAl foam, the pressure drop of air during penetration of the foam was examined. It was found that increased porosity of the material increased the flow of the air through the metallic foam.

## 1. Introduction

Porous intermetallics currently are attracting the attention of researchers due to their mechanical properties and corrosion resistance in oxidative environments and hot temperatures [1]. Aluminides of iron and nickel are among the most frequently-investigated structural intermetallics. Due to their extremely high melting points, traditional methods of foam formation are impossible to apply to these intermetallics. Powder metallurgy provides the opportunity to bypass the limitations and form foams from intermetallics with high melting points. Currently, there are two major approaches to this goal: application of chemically-inert space holders or chemical compounds working as in situ foaming agents. NaCl has been used as a space holder to form porous intermetallics such as TiAl [2], TiAl_3_ [3] or FeAl [4]. According to Ismail et al., NiTi alloy foam was formed with the use of stearic acid and poly methyl methacrylate (PMMA) as space holders [5]. One disadvantage of this method is the required removal of the space holders after the sintering process. Possible remaining sodium chloride that has not leached out would be corrosive, while similarly, organic compounds that neither have leached nor have been burnt may form brittle carbides, spoiling the mechanical properties of the foams. Nevertheless, an elegant and efficient method, based on the use of saccharose as a space holder, has been shown to form titanium foams with pores having a diameter of a fraction of a millimeter [6,7].

An alternative approach also based on space holders consists of sintering with the addition of compounds that decompose during the sintering process and eject gases working as foaming agents. To obtain Ni-Al intermetallic foam, Matsuura et al. applied nickel carbonyl powder as a gas-releasing source in order to receive foam with aluminum powder; carbon oxides were formed in situ [8]. One of the processes based on foaming agents is lost carbonate sintering (LCS). During this process, a carbonate decomposes into metallic oxide and carbon dioxide, which work as an in situ foaming agent. In this way, copper foam was formed with the use of K_2_CO_3_ [9]. Nevertheless, remaining metallic oxides are disadvantageous, due to the hazard of the formation of brittle phases. An effective alternative is the use of chemical compounds that decompose into gaseous products. For this purpose, chemical compounds such as eosine [10], oxalic acid [11], cysteine [12], glycine [12], palmitic acid [13] and cholesteryl myristate [13] were used to form FeAl intermetallic alloy foams. Nevertheless, during sintering and chemical decomposition of the additives, there is a demand for much oxygen. When the access to the oxygen is limited, organic compounds do not only decompose into CO_2_ and H_2_O, but also CO and carbon deposit are formed. During the sintering at high temperature, the carbon deposit may diffuse into the materials and form brittle phases, which should be avoided. Therefore, the application of organic compounds with much oxygen in the molecule is demanded.

Another concept of reactive sintering developed recently is when the starting powders are simultaneously substrates that release gases while decomposing. For example, Kawaguchi et al. reported the formation of porous calcium hexaaluminate from CaCO_3_ and AlOOH powders, in order to release CO_2_ and H_2_O as foaming agents [14]. This method allowed obtaining a material with a porosity of up to 56%. This approach is generally suitable only for ceramic materials, due to the high oxidation states of the metals, Al^3+^ and Ca^2+^ in this case. However, such a reactive sintering employing metals at a high oxidation state allowed also the formation of non-oxide ceramics, like Ti_3_SiC_2_ from TiH_2_, with H_2_, Si and graphite foaming agent donor [15].

Sintering of intermetallic alloys from the Fe-Al binary diagram allows also the use of the thermal explosion (TE) reaction, above 650 °C, which is responsible for the pores foaming [16]. Thus, the addition of a foaming agent at the temperatures over the TE reaction allows one to obtain contributions of both TE and chemical foaming action due to the gas release. A whole range of intermetallics can be formed by using TE or self-propagating high-temperature synthesis (SHS), especially those with high melting points, including some from Ti-Al and Ni-Al [17,18] binary diagrams. However, during these reactions, one has to be aware that metal oxides like NiO may be formed [18].

Metallic foams, due to their morphology, nowadays find numerous applications as implant materials [19], radiation shielding [20], sound absorption [21,22], structural material and crash energy absorbers in the automotive and rail industry [22], heat exchangers [22] and renewable energy harvesting [23], but also bring aesthetics as an added value in building elevations [22]. Therefore, the formation of metallic foam, from material with a high melting point, would allow one to form an element with a highly-developed surface area that could be exploited at high temperatures. At this point, intermetallics, like FeAl, offer numerous potential applications. What is more, FeAl intermetallics have good corrosion performance at high temperatures, which makes this material, in foam form, a potentially interesting material in aggressive, hot environments, like industrial chimneys, where it could work as a filter material. Thus, FeAl intermetallic foam could contribute in emission decreases of smog-forming suspended particles, like PM2.5 and PM10, obtained by coal and hydrocarbon combustion, which is a current problem in Central and Eastern Europe.

In this paper, the formation of FeAl intermetallic alloy by sintering with the additive of tartaric acid is investigated. During the SHS, a comparatively low amount of oxygen is requested for decomposing tartaric acid; therefore, effective foaming should be obtained, with a small amount of side products such as carbon black appearing on the surface.

## 2. Materials and Methods

The starting materials used in this study were: Fe powder, 99% purity, average particle size 100 µm (abcr GmbH, Munich, Germany); Al powder, 99% purity, average particle size <75 µm (Benda-lutz Skawina Sp. z o.o., Skawina, Poland); p.a. (analytical grade purity) tartaric acid (Chempur, Piekary Śląskie, Poland). The following compositions were used: the reference composition (RC), Fe-45Al (at %); RC + 0.5 wt % tartaric acid; RC + 1 wt % tartaric acid; RC + 2 wt % tartaric acid; RC + 5 wt % tartaric acid. Next, the powder mixture was consolidated by uniaxial cold pressing under 700 MPa pressure into cylindrical pellets with a 25-mm diameter and 6-mm height. The sintering process was conducted in a volume-controlled environmental reactor [13] in argon atmosphere at 700 °C for 3 h.

The morphological analysis of the formed foams was carried out with a MA200 optical microscope integrated with NIS-Elements software (Version AR 3.1, Nikon, Tokyo, Japan). Scanning electron microscopy (SEM) imaging and chemical composition analysis (elemental mapping) were carried out with a QUANTA 3D FEG microscope equipped with the X-ray microanalysis device DX4i (FEI, Hillsboro, OR, USA).

The X-ray diffraction (XRD) pattern was taken using the Ultima IV diffractometer (Rigaku, Tokyo, Japan), with Co K radiation (λ = 1.78897 Å) and operating parameters of 40 mA and 40 kV, in the range of 20–130° with a scanning speed of 1°/min and steps of 0.02°.

The pressure drop tests were conducted with home-made equipment, as previously reported [13]. The specimens were discs with a 25-mm diameter and a 10-mm thickness. The discs were inserted into a steel tube to seal the system from the side. Synthetic air was used as the working gas, and pressure drop on the specimen was the measure of permeability. Experiments were conducted in triplicate.

## 3. Results and Discussion

In order to obtain FeAl, the starting composition of Fe-50Al is normally adopted. This was also our first choice during this work, but after reactive sintering, even when 1% of acid was added, the whole sample was falling apart. We suspect that only the FeAl phase was formed, which was more brittle. Application of Fe-45Al allowed us to add more foaming agent, which was the key of the present research.

Typically, much oxygen is required in order to combust organic compounds. On the other hand, when sintering is performed in a closed reactor and the additive is put into the sample, exposure of the in situ foaming agents to the oxygen is limited. Thus, combustion to carbon monoxide or even to carbon black may occur, which would affect the foaming and leave the carbon deposit in place. Carbon was actually found in the specimens when for example phenylalanine and cysteine were used as the foaming agents [12]. In order to fully combust 1 g of cysteine or phenylalanine, 0.87 or 1.62 dm^3^ of oxygen are required, respectively (volume of gases in normal conditions). On the other hand, to combust 1 g of tartaric acid, only 0.37 dm^3^ are required, and a volume of 1.05 dm^3^ of foaming gases is formed, according to the following combustion reaction:

2C_4_H_6_O_6_ + 5O_2_ → 8CO_2_ + 6H_2_O (1)

Furthermore, numerous potentially attractive foaming agents, like crystalline oxalic acid, have water molecules in their crystal structures [11]. What creates a problem is the two-step decomposition of such an additive: at lower temperatures, water is released, and then, at higher temperatures, the proper foaming reaction occurs. Thus, two steps of foaming occur, which could be disadvantageous to the coherence of the foam. However, tartaric acid is anhydrous and additionally poses six oxygen atoms in the molecule, which induces low oxygen consumption per mole of this compound to be combusted (1).

**Figure 1:**
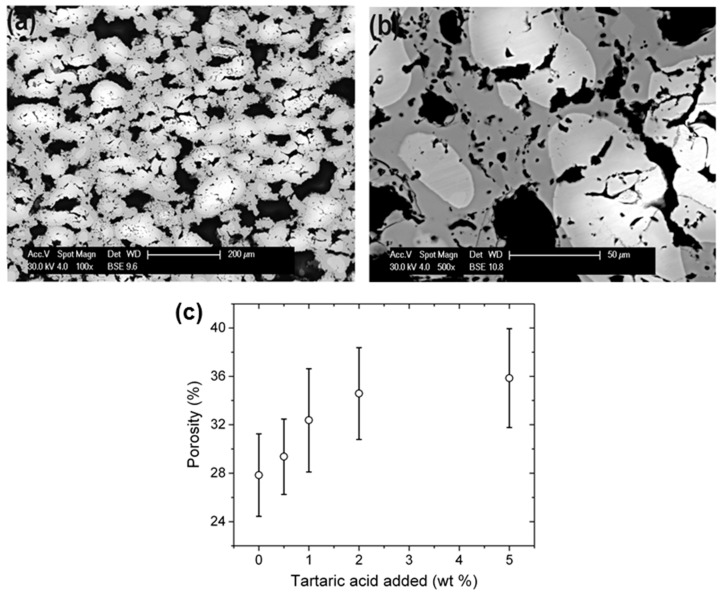
(**a**,**b**) FE-SEM images of Fe-Al intermetallic foams formed by elemental powders sintered with the addition of 5 wt % tartaric acid; (**c**) the porosity of the foams vs. the amount of added tartaric acid.

In the presented experiments, the application of tartaric acid as the additive during the sintering allowed forming metallic foams (Figure 1a,b). Thanks to the use of tartaric acid, there were statistically insignificant differences in pore size between the core and the surface of the specimens. In the core, the average equivalent circle diameter (ECD) was found to be 47 ± 20 µm, while on the surface, it was 35 ± 16 µm. The spread between the ECD between the surface and core of the sample is lower than in the case of the application of other organic compounds [10,11,12,13]. The higher homogeneity of the pore size is ascribed to the lower demand of oxygen for the combustion of the additive during the SHS reaction. On the other hand, the porosity of the Fe-Al metallic foam was up to 36 ± 4% (Figure 1c). Therefore, the porosity obtained was lower than for the case of sintering with eosine Y [10] or oxalic acid [11] (exceeding 45%); however, the distribution of ECD was more uniform. In previous cases [10,11,12,13], the largest pores were in the core of the specimens, whereas smaller, but much more numerous pores appeared close to the specimen surface. For the sintering of elemental powders with tartaric acid, the distribution of pore sizes is obviously more uniform.

**Figure 2:**
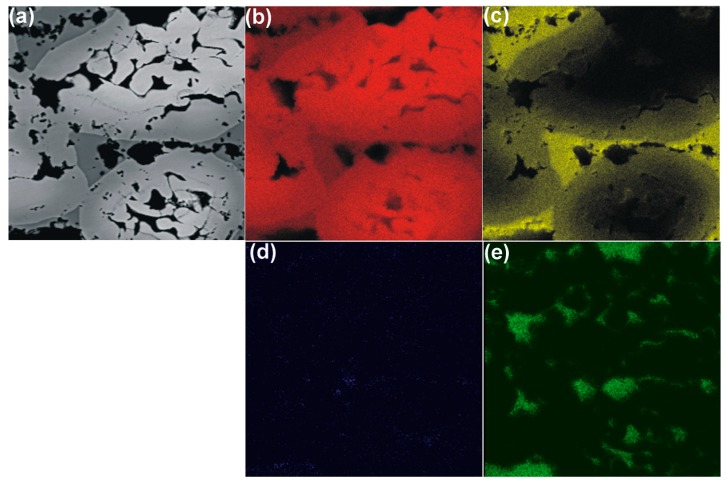
Foams formed via sintering of elemental powders with 5 wt % tartaric acid. (**a**) FE-SEM image; (**b**–**f**) Elemental mapping: (**b**) iron (in red), (**c**) aluminum (in yellow), (**d**) oxygen (in blue) and (**e**) carbon (in green).

**Figure 3:**
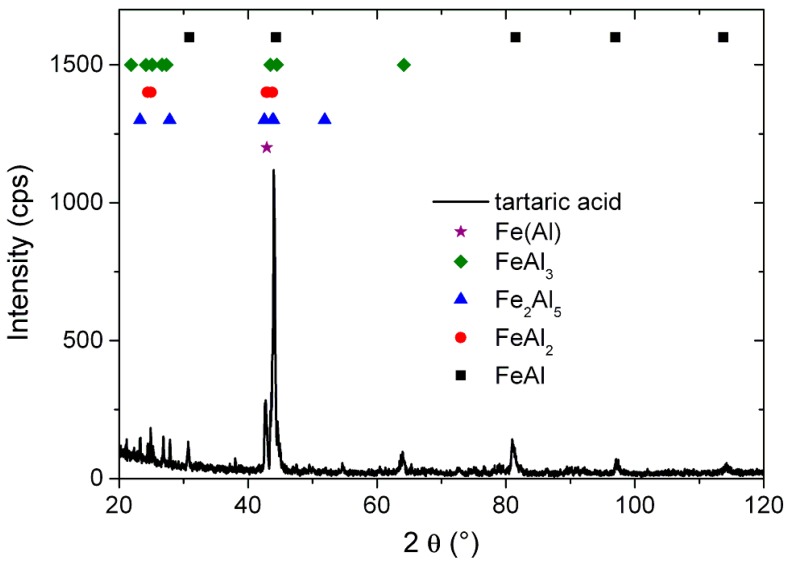
XRD pattern of the sample in Figure 2.

Elemental mapping of the formed foams shows that they are composed of iron and aluminum, and only some traces of oxygen and carbon are present (Figure 2a–e). The traces of carbon were found at the pore bottoms, where the combustion of the organic compound took place. This means that either some unreacted tartaric acid could still be present at the pore bottoms or traces of carbon black were formed in some particular places. Nonetheless, the XRD pattern shows that the formed foams are made of Fe-Al intermetallic alloy (Figure 3).

**Figure 4:**
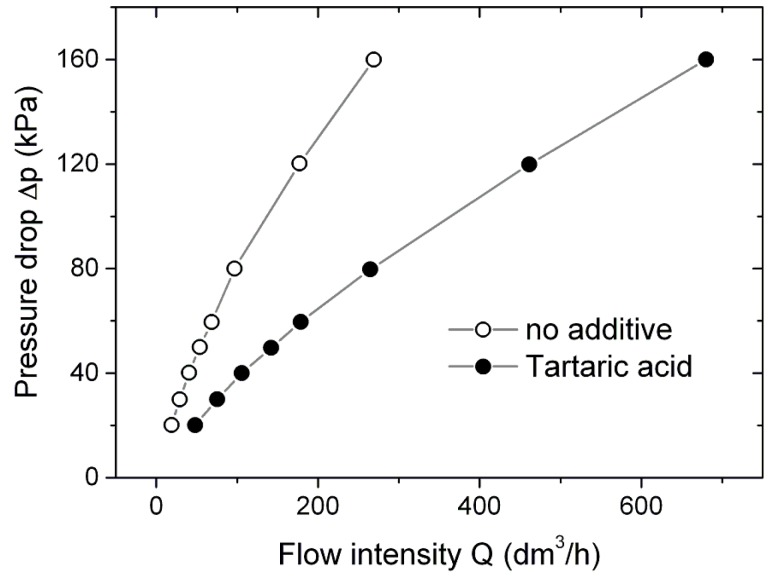
Relationship between pressure drop of air Δp during penetration through the porous FeAl and flow intensity Q, with and without additive.

Metallic foams are attractive for industry as filtration materials. Thus, the formed foams were examined for their permeability. In Figure 4, the pressure drop on the porous barrier is plotted versus the flow intensity. It is found that the foams formed with tartaric acid perform better than those formed without additive. Moreover, their performance is also better than the foams formed with amino acids [12] and comparable to those formed with oxalic acid [11] and eosin Y [10].

## 4. Conclusions

Within the limitations of the present work, the following conclusions can be drawn:

- Sintering of iron and aluminum powders with the addition of tartaric acid allows one to successfully form Fe-Al intermetallic foams.
- Application of tartaric acid as the in situ foaming agent allows one to obtain foams with a more regular distribution of the pores, taking under consideration the surface and core of the specimens.
- The expected performance of the formed foams is promising for future applications of these materials as filters in aggressive environments of hot gases.
